# Evaluation of an in-use chest CT protocol in lung cancer screening - A single institutional study

**DOI:** 10.1177/20584601241256005

**Published:** 2024-07-01

**Authors:** Salma Naimi, Mercy Afadzi Tetteh, Haseem Ashraf, Safora Johansen

**Affiliations:** 1 Health faculty, 60499 Oslo Metropolitan University, Oslo, Norway; 2 Department of Diagnostic Imaging, 60483 Akershus University Hospital, Lørenskog, Norway; 3 Division of Medicine and Laboratory Sciences, University of Oslo, Oslo, Norway; 4 Department of Cancer Treatment, Oslo University Hospital, Oslo, Norway; 5 Health and Social Sciences, Cluster, Singapore Institution of Technology, Singaporee

**Keywords:** Low-dose CT, lung cancer screening, CT dose index, American Association of Physicists in Medicine

## Abstract

**Background:**

Lung cancer is the most common cause of cancer-related death worldwide and therefore there has been a growing demand for low-dose computed tomography (LDCT) protocols.

**Purpose:**

To investigate and evaluate the dose and image quality of patients undergoing lung cancer screening (LCS) using LDCT in Norway.

**Materials and Methods:**

Retrospective dosimetry data, volumetric CT dose index (CTDI_vol_) and dose-length product (DLP), from 70 average-size and 70 large-size patients who underwent LDCT scan for LCS were included in the survey. Effective dose and size-specific dose were calculated for each examination and were compared with the American Association of Physicists in Medicine (AAPM) requirement. For a quantitative image quality analysis, noise, signal-to-noise ratio (SNR), and contrast-to-noise ratio (CNR) were determined for different regions in the chest with two iterative reconstruction techniques, iDose and Iterative Model Reconstruction. Differences in dose and image quality between average-size and large-size patients were evaluated by Independent sample *t* test, and Wilcoxon signed rank test within the same patient group.

**Results:**

The independent sample *t* test revealed significant differences (*p* < .05) in dose values between average-size and large-size patients. Mean CTDI_vol_ and DLP for average-size patients were 2.8 mGy and 115 mGy.cm, respectively, with appropriate increment for the large-size patients. Image quality (image noise, SNR, and CNR) did not significantly differ between patient groups when images were reconstructed with a model based iterative reconstruction algorithm.

**Conclusion:**

The screening protocol assessed in this study resulted in CTDI_vol_ values that were compliant with AAPM recommendation. No significant differences in objective image quality were found between patient groups.

## Introduction

Lung cancer is one of the leading causes of cancer in both men and women worldwide,^1,2^ and while not always the most diagnosed, lung cancer has the highest mortality rate in the world.^3,4^ In 2018, 2100 people died of lung cancer in Norway.^5^ Early detection of lung cancer is currently the most effective way to reduce the total mortality of lung cancer. Patients with lung cancer diagnosed at stage I have a 5-year survival of more than 80% while the total overall survival is 15%–20%.^6^ It is reported that lung cancer screening using low-dose computed tomography (LDCT) results in increased detection and decreased mortality.^7^ In large prospective studies as the National Lung Cancer Screening Trial (NLST) in 2011 and the Dutch-Belgian in a lung cancer screening trial in 2020 confirmed that computed tomography (CT) screening can reduce lung cancer mortality by 20%.^8,9^ A growing number of countries are implementing lung cancer screening programs; however, there is a concern that exposure to the ionizing radiation of LDCT in lung cancer screening might increase the risk of developing solid cancers and leukemia.^1^ Current lung cancer screening (LCS) protocols recommend repeated screening at either annual or biannual frequency for eligible individuals, which can result in over 25 scans over a lifetime.^10–12^ Literature has shown that patient dose varies across regions and countries due to several factors, including CT scanner design, diagnostic protocols, and local-based choices of technical parameters.^12–14^ This will also affect if the image quality is sufficient for detection and measurement for small pulmonary nodules in LCS programs.

There exists a need to implement screening programs for lung cancer around the world.^15,16^ The American College of Radiology (ACR), Society of Thoracic Radiology, and the European Society of Thoracic Imaging have published practice guidelines and technical standards to assist radiologists and medical physicists in developing local CT lung cancer screening protocols.^17–19^ In addition, the American Association of Physicists in Medicine (AAPM) has published a recommended set of lung cancer screening protocols for a range of scanners.^20^ These protocols are based on the experience gained from the NLST study and other screening studies by the working group. These protocols and the ACR guidelines result in a radiation dose (CTDI_vol_) ≤ 3 mGy (≤1.0 mSv) for a standardized patient of 70 kg and a height of 170 cm. However, radiation dose may vary from 0.25 to 5.6 mGy for patient of 50 to 120 kg.^20^ The aim of this study is to investigate and evaluate the dose and image quality of patients undergoing LCS using LDCT at a local hospital in Norway. This will provide valuable information for protocol optimization to further reduce patient radiation dose in the implementation of a future LCS.

## Method and materials

### Patient data

The first lung cancer screening study in Norway started in August 2022. A total of 125,000 Norwegian inhabitants in the age group of 60–79 years were invited to participate in this study using LDCT protocol. Fourteen thousands out of 125,000 agreed to participate and 1,000 of these were selected as participants for the screening study over a 3-year period from 2022 until 2025. Data were collected from the Picture Archiving and Communication Systems, Carestream Vue PACS version 12.2.20105, Philips Healthcare, Best, Netherlands, between February and March 2023.

### Patient selection

Patients between 60 and 79 years, with more than 35 pack years or a PLCOm2012 risk above 2.6%, were eligible for the study. One pack year is one pack of cigarettes (20 cigarettes) a day for one year. PLCOm2012 is a validated risk calculator used for inclusion in lung cancer screening programs. It provides a 6-year risk of developing lung cancer using different which apart from smoking exposure also take into account other factors like family history, BMI, and education level. To ensure a high enough risk group, a threshold of >2.6% was considered.^21^ Cases with severe respiratory motion or prosthetic artifacts were excluded from this study. We retrospectively recorded data from 140 patients who had under gone screening between August and September 2022. Patient weight and height were not available; hence, patients were grouped based on AP chest thickness measured in axial slices at the carina. This was determined by measuring the AP thickness of 42 patients who had under gone contrast enhanced chest pulmonary angiogram examination in December 2022. Body mass index (BMI) was calculated and compared with the thorax AP thickness for each of the 42 patients as shown in Table 1. BMI for average-size and large-size was defined based on the BMI classification described in the AAPM protocols.^20^ Average-size patients were defined with a body mass index ≥18.5 to 24.9 kg/m^2^ and large-size ≥25 kg/m^2^.^22^ The exclusion criteria were patients with a body mass index less than 18.5 kg/m^2^. AP thickness from 20 to 24.9 cm and 25 to 30 cm was therefore defined as average-size and large-size, respectively.

**Table 1.** AP thickness of average-size and large-size patients scanned with a contrast enhanced chest pulmonary angiogram protocol used for inclusion range of AP thickness used in this study.

Number of patient	Average-size body mass index ≥18.5–24.9 kg/m2					Large-size body mass index ≥25 kg/m^2^				
	Weight (kg)	Height (cm)	Age	Body mass index kg/m^2^	AP (cm)	Weight (kg)	Height (cm)	Age	Body mass index kg/m^2^	AP (cm)
1	69	179	73	21.5	22.6	100	178	75	31.6	26.4
2	64	163	69	24.1	21.4	95	178	70	30	27.6
3	70	174	72	23.1	20	119	184	59	35.1	28
4	70	170	56	24.2	23.5	95	172	85	32.1	26.8
5	64	170	73	22.1	21.1	80	166	70	29	25
6	78	177	63	24.9	22.9	105	167	33	37.6	26.7
7	65	168	78	23	20.4	102	170	68	35.3	26.6
8	65	166	67	23.6	21.6	125	169	32	43.8	29.5
9	78	178	88	24.6	22.6	117	164	48	43.5	25.7
10	77	165	58	21.3	20.4	90	168	65	31.9	25.8
11	76	186	79	22	21	88	180	80	27.2	25.4
12	54	154	89	22.8	20.9	52	168	98	34.7	25.2
13	64	175	77	20.9	22.4	114	172	39	38.5	25.9
14	60	160	57	23.4	20.5	104	170	43	36	25.1
15	60	160	84	23.4	23.5	94	186	60	27.2	25.3
16	70	176	64	22.6	22.9	97	187	68	27.7	25.3
17	69	170	80	23.9	23.8	100	187	78	28.6	25.9
18	56	161	75	21.6	22.7	156	195	59	41	29.2
19	78	183	59	23.3	23.9	130	186	48	37.6	29.6
20	80	181	78	24.4	24.5	92	177	61	29.4	26.1
21	73	175	74	23.8	22.3	82	161	83	31.6	25.2

### CT acquisition parameters

CT examinations of the thorax were performed using a Brilliance iCT 256 scanner (Royal Philips, 2012, Amsterdam, The Netherlands) in supine position with arms raised above the shoulders to prevent artifacts. The patients were provided instructions to prevent any voluntary motion and to cautiously follow the breath-hold instructions. The scanner characteristics and protocol parameters are shown in Table 2. Raw data was reconstructed with two iterative reconstruction techniques iDose and Iterative Model Reconstruction (IMR). IDose is a hybrid or statistical iterative reconstruction algorithm whereas IMR is a model based iterative reconstruction algorithm.^23^

**Table 2.** Characteristics of the CT scanners and acquisition parameters included in the survey.

Manufactures	Philips
Model	Brilliance iCT 256
Install year	2012
Detector configuration (mm)^a^	128 × 0.625
Iterative reconstruction (level)	iDose (3) and IMR (1)
Tube voltage	120
AEC settings	Min/max mA25/200, DRI^b^ 9
Acquired/recon slice (mm)	0.9/0.45
Pitch	1.171
Rot time (s)	0.33
Kernel	iDose: Y-sharp / IMR: Soft tissue
Window width/window level (HU)	iDose: 1600/-600 / IMR: 400/40
Software version	4.1.7

^a^Notation of the detector configuration: the number of active detector rows x the detector element thickness.

^b^Dose right index.

### Radiation dose assessment

Volume computed tomography dose index (CTDI_vol_) and dose-length product (DLP) values were collected for each examination retrospectively. The estimated effective dose was calculated as DLP multiplied by a k-factor of 0.014 mSv^*^mGy^−1^ cm^−1^ for the chest.^24^ Size-specific dose estimate (SSDE) was calculated as described in the AAPM Report 204.^25^ Thus, SSDE was calculated for each examination based on the recorded CTDI_vol_ values and the corresponding AP chest thickness conversion coefficient.^24^

### Objective image analysis

**Figure 1.**
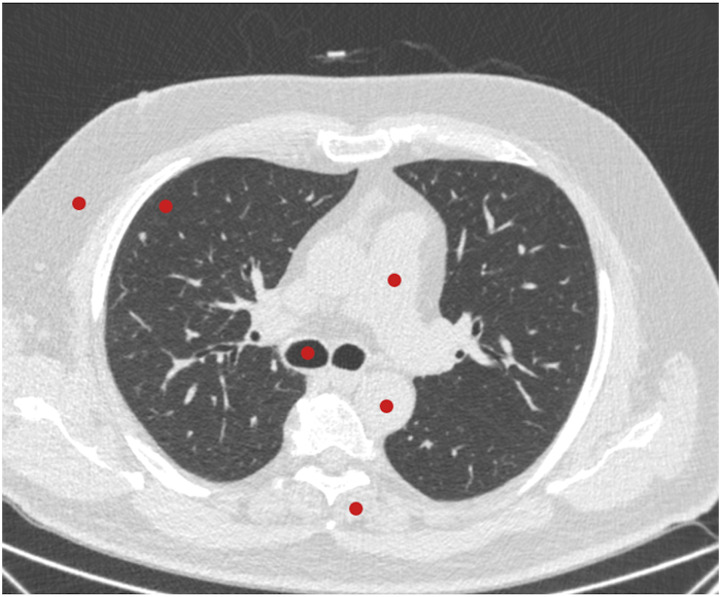
Placement of the regions of interest for an objective image quality assessment: the subcutaneous fat, peripheral lung parenchyma, air within the trachea, truncus pulmonalis, aorta descendent, and paravertebral muscle.

Standardized 30-mm-diameter circular regions of interest (ROI) were used to record signal and noise, which represented mean attenuation value and standard deviation (SD) in Hounsfield units (HU). The ROIs were placed in the axial slices at the level of tracheal bifurcation in the subcutaneous fat, peripheral lung parenchyma, air within the trachea, truncus pulmonalis, aorta descendent, and paravertebral muscle,^26^ as shown in Figure 1. Moreover, the signal-to-noise ratio (SNR) was calculated in all ROIs as follows^27^:

$$\text{SNR}=\frac{{HU}_{1}}{{SD}_{1}}$$

The paravertebral muscle measurements were used as the background attenuation to calculate contrast-to-noise ratio (CNR) as follows^27^:

$$\text{CNR}=\frac{\left({HU}_{1}-{HU}_{2}\right)}{{SD}_{2}}$$

where HU_1_ is the attenuation value measured in peripheral lung parenchyma or aorta descendent or truncus pulmonalis and HU_2_ and SD_2_ are the attenuation value and noise measured in the paravertebral muscle.

### Statistical analysis

Data were recorded in Excel (Microsoft Office 2010) and were analyzed with the Statistical Package for the Social Sciences software version 18.0 (IBM Corp.). The Kolmogorov–Smirnov test was performed to determine whether data were normally distributed. Descriptive statistical analysis of the collected dosimetry parameters, CTDI_vol_, DLP, SSDE, image noise, SNR, and CNR values was performed for each patient to determine the mean and standard deviation. Independent sample *t* test was used to compare the differences between average-size and large-size patients. Two-way ANOVA was used to investigate gender differences between the same size groups. To compare the difference in image quality within the same patient group, the Wilcoxon signed ranks test was used. The *p* value <.05 was considered as statistically significant.

### Ethical considerations

This study was approved by Data Protection Office/institutional review board at the involved hospital defined as a quality assurance study. The need for informed consent for the use of existing CT scan images, including raw data, was waived. No sensitive patient data was recorded in the study.

## Result

### Patient selection

Of 140 patients, 78 (56%) were men and 62 (44%) were women, and the mean age of the participants was found to be 70 years (age range, 62–81) years (Table 3).

**Table 3.** Basic characteristics of the participants.

Characteristics	Average-size *n* (%)	Large-size *n* (%)
Number of study participants	70 (50)	70 (50)
Gender		
Male	22 (30)	56 (80)
Female	48 (70)	14 (20)
Age (years), median ± SD	70 ± 5	69 ± 5
Range (years)	62 - 81	62 - 81

**Figure 2.**
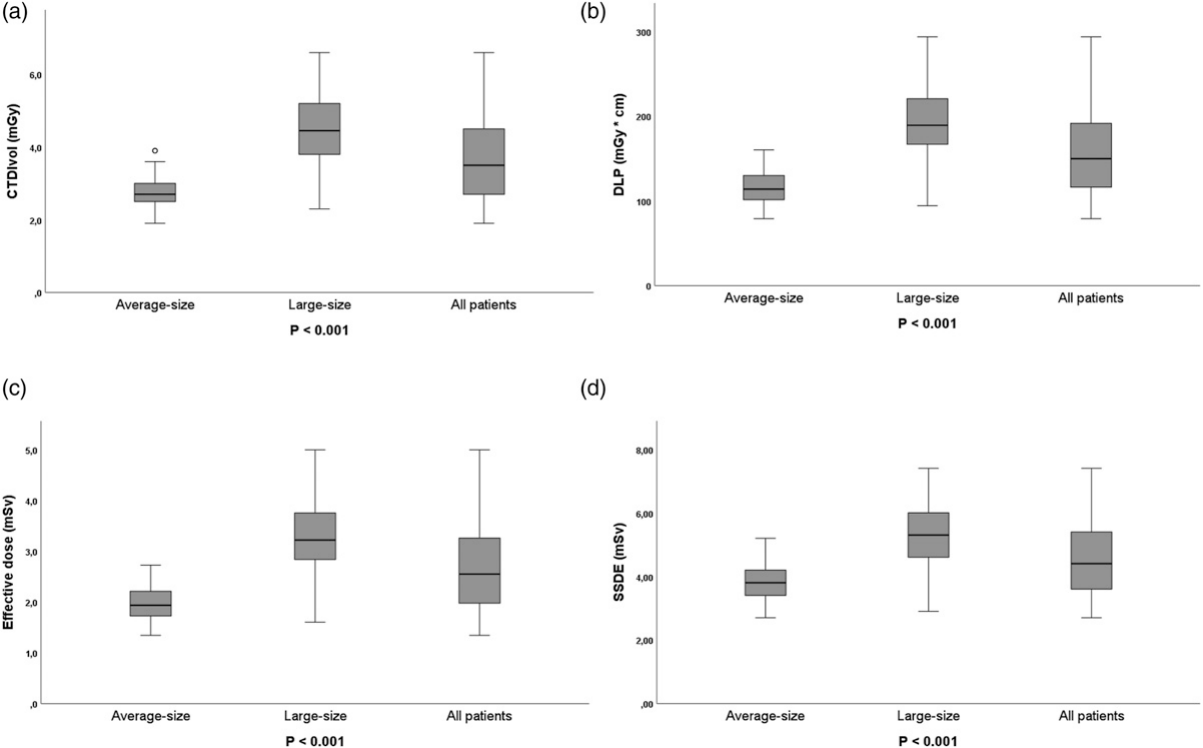
Box plot showing (a) CTDI_vol_ = volume CT dose index, (b) DLP = dose-length product, (c) effective dose, and (d) SSDE = size-specific dose estimate of lung cancer screening using low-dose computed tomography. Box plot midlines indicate medians, outer lines indicate 25th and 75th percentiles, respectively, and whiskers indicate the range of data points excluding outliers represented by blank circle. *p* < .05 indicates statistical significance between average-size and large-size.

Figure 2 shows box plots of CTDI_vol_, DLP, effective dose, and SSDE categorized according to patient’s size. The mean CTDI_vol_ of average-size patient was found to be 2.8 ± 0.48 mGy (range, 1.9–3.9) which is significantly lower (*p* < .001) than that of the large-size patients (4.5 ± 0.96 mGy; range, 2.3–6.6 mGy). Nonetheless, mean CTDI_vol_ for the entire patient group was found to be 3.62 mGy. No significant difference was observed with average scan length of the two patient groups (*p* = .204). Consequently, mean DLP of the large-size group (193.6 ± 42.37 mGy∙cm; range, 94.2–293.5) was significantly (*p* < .001) higher than that of the average-size patients (115.22 ± 21.45 mGy∙cm, range, 79–160.2). The same trend was seen for the effective dose and SSDE data, where effective dose and SSDE of the large-size patients were 3.3 ± 0.72 mSv (range, 1.6–5) and 5.3 ± 1.03 mGy (range, 2.9–7.4) and that of average-size patients were 2 ± 0.4 mSv (range, 1.3–2.7) and 3.8 ± 0.67 mGy (range, 2.7–5.2), respectively (Figure 2). No correlation between gender and dose was observed in this study. No statistically significant difference was found between the CTDI_vol_ (*p* = .28), DLP (*p* = .32), SSDE (*p* = .31), and effective dose (*p* = .3) when comparing the dose in men versus women within the same size group.

**Figure 3.**
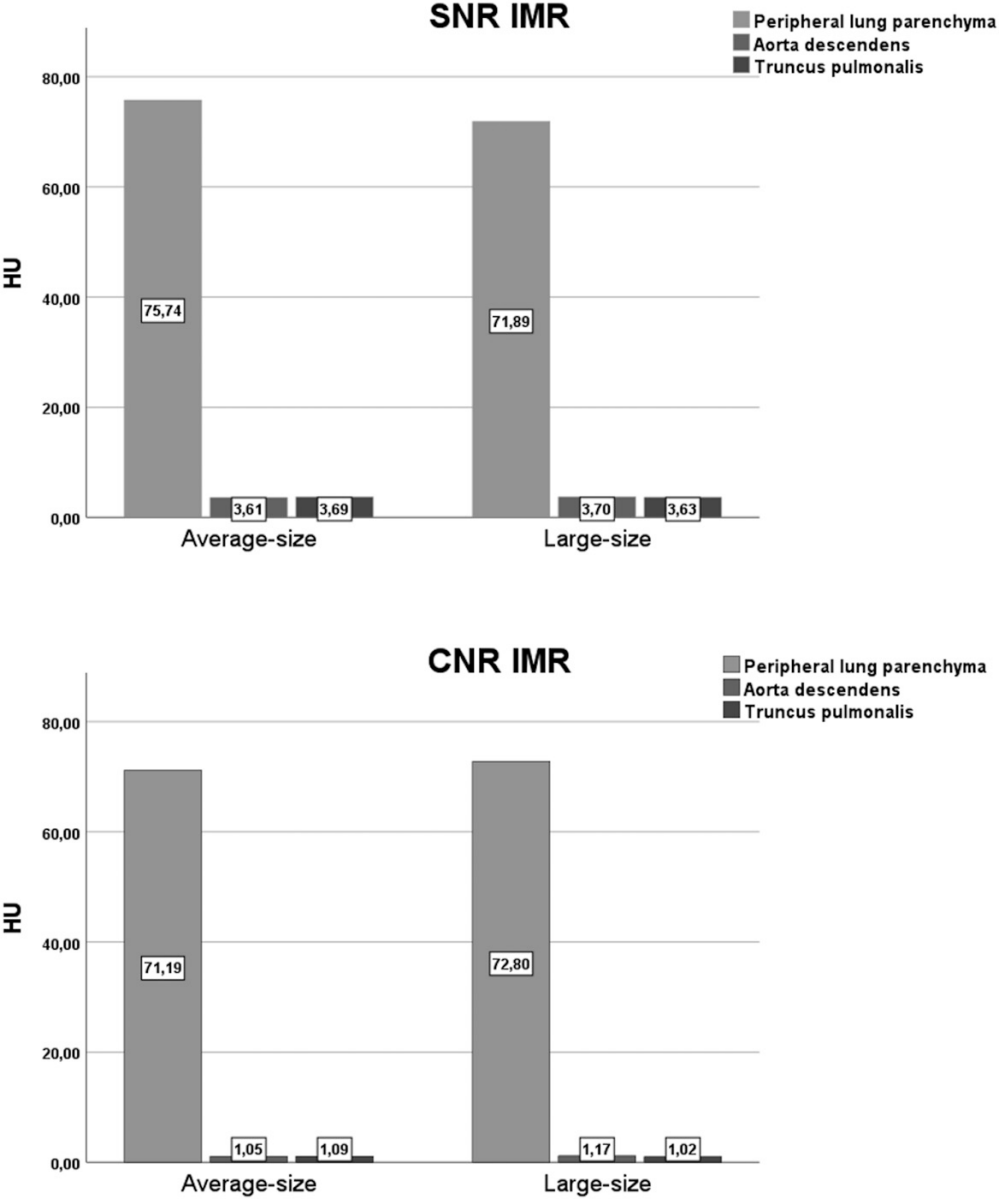
Bar chart showing (a) SNR = signal-to-noise ratio and (b) CNR = contrast-to-noise ratio in mean for average-size and overweight. HU = Hounsfield units.

The objective image analysis results are presented in Table 4. The image noise in the air within the trachea and peripheral lung parenchyma did not significantly differ between average-size and large-size for iDose, except for subcutaneous fat (*p* = .009), truncus pulmonalis (*p* = .044), aorta descendent (*p* = .015), and paravertebral muscle (*p* = .003) that had a significant difference. Image noise, SNR, and CNR of images reconstructed with IMR did not significantly differ between average-size and large-size for all tissue types (Figure 3). The same trend was seen for images reconstructed with iDose, except CNR of the peripheral lung parenchyma (*p* = .001). As expected, significant difference was found for image noise, SNR, and CNR of iDose and IMR images within the same patient group for all tissue types, where IMR improves the image quality of LDCT more than hybrid algorithm iDose (*p* < .001).

**Table 4.** Results of the objective image quality assessment: image noise, SNR, and CNR. Results are presented as median ± SD.

Parameter	iDose		IMR		iDose	IMR
	Average-size	Large-size	Average-size	Large-size	Difference between average-size and large-size *p*-value	Difference between average-size and large-size *p*-value
Image noise (HU)						
Air within the trachea	27.21 ± 3.81	27.49 ± 4.48	13.16 ± 2.18	13.09 ± 2.69	0.700	0.861
Peripheral lung parenchyma	24.93 ± 4.50	25.46 ± 3.24	12.33 ± 12.07	13.11 ± 2.72	0.441	0.059
Subcutaneous fat	30.53 ± 5.34	33.04 ± 5.89	11.38 ± 1.44	11.30 ± 1.62	0.009	0.769
Truncus pulmonalis	34.25 ± 5.60	36.31 ± 5.89	12.60 ±1.85	12.82 ± 1.15	0.044	0.395
Aorta descendent	35.76 ± 6.21	38.42 ± 6.53	12.43 ± 1.31	12.64 ± 1.67	0.015	0.412
Paravertebral muscle	36.83 ± 5.90	39.59 ± 5.05	14.26 ± 4.05	13.35 ±2.34	0.003	0.106
SNR						
Peripheral lung parenchyma	37.42 ± 7.19	36.06 ± 5.46	75.74 ± 14.32	71.89 ± 15.13	0.212	0.124
Truncus pulmonalis	1.40 ± 0.36	1.33 ± 0.30	3.61 ± 0.72	3.70 ± 0.69	0.667	0.422
Aorta descendent	1.31 ± 0.25	1.29 ± 0.30	3.69 ± 0.69	3.63 ± 0.53	0.243	0.601
CNR						
Peripheral lung parenchyma	26.23 ± 4.54	24.09 ± 3.63	71.19 ± 18.96	72.81 ± 13.41	0.001	0.561
Truncus pulmonalis	0.42 ± 0.31	0.37 ± 0.32	1.05 ± 0.82	1.17 ±0.96	0.199	0.455
Aorta descendent	0.41 ± 0.30	0.37 ± 0.27	1.09 ± 0.81	1.02 ± 0.75	0.206	0.587

*p* < .05. HU = Hounsfield unit, SD = standard deviation, CNR = contrast-to-noise ratio, SNR = signal-to-noise ratio.

## Discussion

In this study, the impact of a LDCT protocol on dose and image quality in both average-size and large-size patients was investigated. The results of this study showed a significantly higher radiation dose for large-size patient group compared to average-size. However, no significant difference in objective image quality was observed between the two patient groups.

Even though the risk of radiation-induced cancer is low compared to the benefits of LCS, it is important to keep all doses as low as possible, especially for patients undergoing repetitive examinations.^28^ According to the ACR and AAPM recommendation, LDCT must be performed with a CTDI_vol_ of ≤3.0 mGy for a standard-sized patient.^18,20^ In the present study, the mean CTDI_vol_ for the average-sized patient was found to be 2.8 mGy, which is well within the recommendation. For protocols that use AEC methods, the tube output will be dependent on the patient’s anatomy. Our results also reveal that the Philips AEC (DoseRight Z + 3D) used in this study allowed the tube output to be adjusted according to patient size. Thus, mean CTDI_vol_ used for the large-size patients was significantly higher than that of the average-sized patients. Other researchers have also reported CTDI_vol_ < 3 mGy for average-size patients with appropriate reduction and increment for patients with lower and higher BMIs.^29,30^ No statistically significant difference was found between the radiation dose (CTDI_vol_, DLP, SSDE, and effective dose) related to gender within the same size group. The results are in agreement with doses reported by Chu et al^31^ confirming that dose variation is dependent on patient size and not gender.

Mean CTDI_vol_ observed in this study for average-sized and large-size patients (2.8 and 4.5 mGy, respectively) was higher than what AAPM proposes for a Philips Brilliance iCT 256 scanner (2.7 and 3.6 mGy, respectively). Additionally, mean DLP and ED observed for average-size patients in this study were 115 mGycm and 2 mSv, respectively, which are higher than what AAPM proposes for a standard-sized patient (≤75 mGycm and ≤1.0 mSv, respectively).^20^ These discrepancies might be due to difference in scan parameters and scanning techniques. Also, difference in software version can affect the radiation dose according to the vendor. Besides, research has shown that small differences in scan techniques even with the use of AEC can lead to variation in radiation doses for the same patients.^32^ Changes in the table height, scan length, and small differences in the patient’s positioning may also affect the patients’ radiation dose, according to research.^32,33^ For low-dose CT examination, the desired coverage is from the apex to the lung bases. However, technologist will often add a “safe margin” to the scan length to assure complete coverage, which may increase the DLP.^33^ Hence, scan length should be adapted strictly to the clinical indication for each individual patient and must be limited to the area of interest in order to avoid unnecessary radiation dose to the patient.^34,35^ This suggest that there is potential for dose reduction which implies the protocol will benefit from optimization.

In lung screening, it is critically important to ensure high confidence in the detection and volumetric measurements of small lung nodules.^36^ The assessment of small lung nodules does not require high CNR but good spatial resolution because of the inherent high contrast present in the chest.^37^ Thus, the evaluation of the lung parenchyma can tolerate high noise. Therefore, it is important that careful attention be paid to the selection of exposure parameters in order to achieve the desired level of image quality.^36^ Our results revealed no significant differences in image noise between patient sizes when images where reconstructed with IMR and 0.9 mm slice thickness. The reconstructed slice thickness is a critical determinant for the detection and volumetric measurement accuracy of small nodules as well as the ability to reconstruct images in multiple planes. Consequently, thinner slice thickness increases spatial resolution and decreases the partial volume effect even though it increases image noise.^38,39^ In the present study, slice thickness of 0.9 mm was used for both lung and soft tissue reconstructions. Study findings have shown greater variability and inaccuracy in volume determination of small nodules compared with large nodules with different slice thicknesses.^38–40^ Winer-Muram and colleagues^41^ found that an increase in slice thickness leads to an increase in overestimation of nodule volume. Our data indicates that MBIR (IMR) improves the image quality of LDCT more than hybrid algorithm (iDose) which is in accordance with previous studies.^42^

The present study has some limitations that need to be addressed. Thus, patient’s weight and height were not available for this study, making it impossible to calculate BMI of the patients. Hence, patient grouping was done based on AP diameter measured in axial slices at the carina and compared with that of previous studies where weight and height were recorded. The study would have benefited from a qualitative image quality assessment; however, this is a retrospective study so all images had been evaluated by one or two radiologist and image quality was considered clinically adequate. Finally, the study was carried out at a single institution. However, our findings are generally in line with previous studies and therefore may well be valid to other centers. In conclusion, this study showed that the CTDI_vol_ values from a lung cancer screening program were within the AAPM recommended dose for average-size patients. It also showed the use of AEC adjusted the dose according to patient size without affecting image quality. However, the protocol might benefit from optimization of scan parameters in order to reduce the radiation dose further.
